# Evaluation of leishmanicidal activity and cytotoxicity of *Ricinus communis* and *Azadirachta indica extracts from* western Kenya: in vitro and in vivo assays

**DOI:** 10.1186/s13104-015-1605-y

**Published:** 2015-11-05

**Authors:** Bernard N. Jumba, Christopher O. Anjili, Judith Makwali, Johnstone Ingonga, Rose Nyamao, Sylvia Marango, Joseph K. Choge, Christopher Khayeka-Wandabwa

**Affiliations:** Department of Biological Science (Parasitology), University of Eldoret, P.O Box 1125-30100, Eldoret, Kenya; Centre for Biotechnology Research and Development (CBRD), Kenya Medical Research Institute (KEMRI), P.O Box 54840-00200, Nairobi, Kenya; Zoology Department, Jomo Kenyatta University of Agriculture and Technology (JKUAT), P.O. Box 62000-00200, Nairobi, Kenya; Institute of Tropical Medicine and Infectious Diseases-KEMRI (ITROMID-KEMRI), Jomo Kenyatta University of Agriculture and Technology (JKUAT), P.O. Box 62000-00200, Nairobi, Kenya; University of Kabianga, P.O. Box 2030-20200, Kericho, Kenya; African Population and Health Research Center (APHRC), P.O. Box 10787-00100, Nairobi, Kenya; Applied Science Department, Sigalagala National Polytechnic, P. O. Box 2966, Kakamega, Kenya; Department of Medical Laboratory Sciences, Masinde Muliro University of Science and Technology, P.O Box 190, Kakamega, 50100 Kenya

**Keywords:** Leishmaniasis, *Ricinus communis*, *Azadirachta indica*, *Leishmania major*, Pentostam, Amphotericin B, Combination therapy, 50 % inhibitory concentration, Toxic, Efficacy, In vitro and in vivo

## Abstract

**Background:**

Despite advances to targeted leishmanicidal chemotherapy, defies around severe toxicity, recent emergence of resistant variants and absence of rational vaccine still persist. This necessitates search and/or progressive validation of accessible medicinal remedies including plant based. The study examined both in vivo and in vitro response of *L. major* infection to combined therapy of *Ricinus communis* and *Azadirachta indica* extracts in BALB/c mice as the mouse model. A comparative study design was applied.

**Results:**

BALB/c mice, treated with combination therapy resulted in significantly (*p* < 0.05) larger reduction of lesion than those treated with monotherapies. The spleno-somatic index was found to be significantly low with combination therapy than monotherapies. Antiparasitic effect of *A. indica* and *R. communis* on amastigote with a 50 % inhibitory concentration (IC_50_) was of 11.5 and 16.5 µg mL^−1^ respectively while combination therapy gave 9.0 µg ml^−1^ compared to the standard drugs, Pentostam and amphotericin B which had an IC_50_ of 6.5 and 4.5 µg ml^−1^ respectively. Optimal efficacy of *A. indica* and *R. communis* was 72 and 59.5 % respectively, combination therapy gave 88 %, while Pentostam and amphotericin B had 98 and 92 % respectively against amastigotes. Against promastigotes *A. indica* and *R. Communis* gave an IC_50_ of 10.1, 25.5 µg mL^−1^ respectively, while combination, 12.2 µg mL^−1^ against 4.1 and 5.0 µg ml^−1^ for Pentostam and amphotericin B respectively. The optimal efficacy of the compounds against promastigotes was 78.0, 61.5 and 91.2 % (*A. indica, R. communis* and *A. indica* + *R. communis* respectively) against 96.5 and 98 % for Pentostam and amphotericin B respectively. The concentrations at optimal efficacy were significantly different (*p* < 0.05) among the test compounds. An evaluation of the IC_50_ values of the combination therapies clearly reveals synergistic effects.

**Conclusion:**

Combination therapy of *A. indica* and *R. communis* had best antileishmanial activity than the monotherapies. The active ingredients of both *R. communis* and *A. indica* need to be fractionated, and studied further for activity against *Leishmania* parasites.

## Background

In many tropical and subtropical developing countries, protozoan parasites are amongst the most common infectious agents and have serious consequences for socio-economic development [1, 2]. The World Health Organization (WHO) considers leishmaniasis to be one of the most serious parasitic diseases and the World Health Assembly has advocated a concertation for its control [3]. Due to species differences in tissue tropism, virulence and their interaction with the host’s immune system, infection by leishmaniasis can result in a variety of clinical manifestations ranging from single self-healing ulcers in cutaneous forms to life threatening visceral infections [4, 5]. The pentavalent antimonials sodium stibogluconate and meglumine antimoniate alongside their generics have been the first-line treatment for leishmaniases in many areas for decades [6–8]. Albeit, antimonials are considered to be toxic with frequent, sometimes life-threatening, adverse side effects. Patients under the age of 2 or aged ≥45 with signs of advanced disease and/or severe malnutrition are at higher risk of death during therapy owing to drug toxicity, slowness of drug action, infection complications or a combination of these factors [9–11]. Although there is still a need for more research and development (R&D) to improve the drug pipeline for leishmaniasis [12], some alternatives have become available in recent years. The most pressing research needs for leishmaniasis control are the search for alternative and cost effective drugs for oral, parenteral or topical administration in shorter treatment cycles, and identification of mechanisms to facilitate access to existing control measures, including health-sector reform in some developing countries. These will help mitigate the neglected tropical disease (NTDs) associated chronic vicious cycle of poverty in sub-Saharan Africa and other contexts with high prevalence.

The castor oil plant, *Ricinus communis* is a species of flowering plant in the spurge family, *Euphorbiaceae*. It belongs to a monotypic genus, *Ricinus*, and sub-tribe, *Ricininae*. Alcoholic extract of the leaf has been shown to be hepatoprotective in rats [13]. Methanolic extracts of the leaves of *R. communis* have shown antimicrobial properties [14]. The pericarp of castor bean showed central nervous system effects in mice at low doses. Antihistamine and anti-inflammatory properties have been found in ethanolic extract of *R. communis* root bark [15]. The neem tree, *Azadirachta indica, A. Juss* (*Meliaceae*) is an Indian tree that has many useful compounds that act as insecticide. It has also been documented to have anti-leishmanial effect on *L. donovani* [16]. However, little information is available on efficacy of combination therapy of *A. indica* and *R. communis*.

Considering conventional targeted leishmanicidal chemotherapy options are out of reach for most rural communities in developing countries, alternatives are bound to be sort or incorporated in rational management of the complication. Evidently, many of these populations and individually held knowledge by the traditional health practitioners (THP) know a lot about medicinal flora that can cure a diversity of diseases while combination of medicinal remedies by the herbalists/self-prescription is now a common occurrence [17–20], but due to a number of protocols required, their effective use remain speculative. There are limited studies available interrogating the anti-parasite properties on synergistic, antagonistic or toxicity effect of combined medicinal therapies in various medical conditions. Furthermore, efficacy of plant extracts is known to be affected by among other things; location, amount of active compounds in the plants, extraction procedure and species of organism under study; which makes it very difficult to generalize the anti-parasitic/pathogenic properties of many plant species. In light of this, especially in the tropical regions where there are large forested land under these plants, the aim of this study was to assess response of *Leishmania major* to combined therapy of *R. communis* and *A. indica* extracts in BALB/c mice.

## Methods

### Experimental design

The in vivo and in vitro studies were carried out using a comparative study design. The efficacy and toxicity of samples were compared with those of Pentostam (GlaxoSmithKline, UK) and amphotericin B [Fungizone™, X-Gen Pharmaceuticals (US)]. In vivo studies were further subjected to a complete randomized block design. The results were compared to determine the efficacy of the test samples against the known standard drugs for treating leishmaniasis.

### Anti-leishmanial plants and extracts

The plant extracts were obtained from the *A. indica* and *R. communis*. Leaves of *A. indica* and *R. communis* were collected from Kakamega forest Western region of Kenya with the assistance of a plant taxonomist and their voucher specimens were deposited at the East African Herbarium, National Museums of Kenya, Nairobi, Kenya (*Ricinus communis* TFm13 and *Azadirachta indica* TFm23). The plant extracts were processed according to the method of Kigondu et al. [21]. The plant parts were chopped into small pieces; air dried at room temperature (25 °C) for 14 days and pulverized using a laboratory mill (Christy & Norris Ltd., Chelmsford, England). 1 kg of each powder was soaked in absolute methanol for 3 days to extract compounds. The extract was filtered, dried with Na_2_SO_4_ and the solvent removed under vacuum in a rotary evaporator at 30–35 °C. For aqueous extraction, 100 g of ground material in 600 ml of water was placed in a water bath and maintained at 60 °C for 2 h. This filtrate was freeze dried (using a Freeze Dryer, Edwards freeze dryer Modulyo), weighed and stored at −20 °C until required for use. DMSO was used in all the drug formulations because it has been reported to increase drug penetration [22]. These methanolic extracts were used for anti-leishmanial testing. The plant yields for *A. indica* and *R. communis* were 15–18 % w/w and 10–15 % w/w of plant leaves respectively.

### Mice and parasites

Female 8 week old BALB/c mice weighing 20 ± 2 g were used in the experiment. The animals were obtained from Kenya Medical Research Institute (KEMRI) animal breeding facility, Nairobi-Kenya. The animals were moved into the experimental room for acclimatization one week before the start of the experiments. The mice were housed in 15 cm × 21 cm × 29 cm transparent plastic cages. They were fed with pellets (Mice pellets UNGA^®^ feeds) and water ad libitum.

*Leishmania major* (strain IDUB/KE/83 = NLB-144) which was originally isolated in 1983 from a female *P. duboscqi* collected near Marigat, Baringo County Kenya was used [23]. The *L. major* strain was maintained by serial passage in BALB/c mice to maintain virulence. An aspirate isolate from the footpad of infected BALB/c mouse was cultivated in Schneider’s *Drosophila* insect medium (Sigma, Saint Louis, USA), supplemented with 20 % heat inactivated foetal bovine serum (FBS) (Cultilab, Campinas, SP, Brazil), 500 µg/ml penicillin, 500 µg/ml streptomycin and 250 µg/ml 5-fluorocytosine arabinoside (all from Gibco, Grand Island, NY, USA) [24, 25]. Promastigotes were incubated at 25 °C grown to stationary phase to generate infective metacyclic forms at 6th day of culture. Promastigotes in the medium were counted with a hemocytometer (Improved Double Neubauer) (Pharmacia-GE Healthcare, Uppsala, Sweden) with a Nikon optiphot optical microscope at 40× magnification.

### In vitro studies

#### Cytotoxicity assay

In vitro cytotoxicity assay was carried out following a modified rapid colorimetric assay as previously described by Mosmann [26]. Vero cells were cultured and maintained in Eagle’s Minimum Essential Medium (MEM) supplemented with 10 % FBS. The cells were cultured at 37 °C in 5 % CO_2_ for 24 h, harvested by trypsinization, pooled in a 50 ml vial and 100 µl cell suspension (1 × 10^6^ cells/ml) put into 2 wells of rows A-H in a 96-well micro titer Nunc-Immuno™ (MaxiSorp™ Surface) plate, the medium aspirated off and 150 µl of the highest concentration of the *A. indica* and *R. communis* added into the same row and serial dilution. The controls used were cells with no extract and medium alone. MTT (3-4, 5-dimethgylthiaol-2-yl-2, 5-diphenyltetrazolium bromide) reagent (10 µl) was added into each well and the cells incubated for 4 h. After which, the medium together with MTT were aspirated off. Dimethylsulfoxide (DMSO) (100 µl) was added and the plates shaken for 5 min. The absorbance was measured for each well at 562 nm using a micro-titre plate reader [27].

#### Minimum inhibitory concentration (MIC) and anti-promastigotes assay

*Leishmania major* promastigotes (10^6^ parasites/ml) was incubated at 26 °C for 120 h in fresh media (brain heart infusion medium), supplemented with 10 % FBS in the absence or presence of serial concentrations (100, 50, 25, 12.5, 6.25 and 3.125 µg/ml) of the extracts. Cell growth was determined daily by assessment of visible turbidity. The MIC was considered as the lowest concentration of each substance used that inhibited more than 99 % of *L. major* growth in vitro.

Promastigotes were incubated in 24-well plates in the presence of serial concentrations of the extracts for 120 h. Aliquots of parasites were then transferred to 96-well Nunc-Immuno™ (MaxiSorp™ Surface) microtiter plate, incubated at 27 °C in 5 % CO_2_ for 24 h, then 100 μl of highest concentration of the extract was added and serially diluted, then incubated further at 27 °C for 48 h. The controls used were promastigotes with no extract and medium alone. MTT reagent (10 μl) was added and incubated for 4 h, then the medium together with MTT was aspirated off; DMSO (100 μl) was added and the plates were shaken gently for 5 min. The absorbance was measured for each well at 562 nm using a micro-titer plate reader [26].

#### Macrophage cultures for anti-amastigote assay and determination of nitric oxide

Four days after intraperitoneal injection of female BALB/c mice (8–12 week old; Kenya Medical Research Institute (KEMRI) animal breeding facility, Nairobi-Kenya) with 4 % Brewer’s thioglycolate broth (Difco, Inc., Detroit, MI), peritoneal macrophages were harvested with Phosphate-buffered saline (PBS) and processed as described [28, 29]. The derived macrophages were infected with *L. major* stationary phase promastigotes at a 6:1 parasite/macrophage ratio. Uninfected macrophages were used as negative controls. Infected macrophages were incubated at 37 °C in 5 % CO_2_ for 4 h. After incubation, the remaining extracellular parasites were removed by gentle washing and the cultures incubated in RPMI (JRH Biosciences, Lenexa, KS) for 24 h. Subsequently, treatment of infected macrophages with the *R. communis* and *A. indica* was done as earlier described [30, 31].The medium replenishment was for 3 days (24, 48 and 72 h time points). Pentostam and amphotericin B were used as a positive control drugs for comparison of parasite inhibition. On day four, the wells were washed twice with normal saline and the reaction was stopped by adding stop solution, then incubated for 2 h, washed twice with normal saline and fixed with methanol then stained with Giemsa stain. The infection levels were determined by counting the percentage of infected cells and the number of amastigotes per 100 macrophages [30]. This analysis was performed by two independent observers who were blinded to the experimental conditions. After each time point, supernatants were harvested and stored at −70 °C for analysis of nitric oxide (NO) production. Nitrite (NO_2_^−^) accumulation in the cell culture supernatants was used as an indicator of NO production and was determined by a standard Griess reaction [28, 29, 32].

#### In vivo; experimental infections setup, treatment, lesion measurement and parasite burden

For in vivo settings, the sample size was calculated using the resource equation method [33]. 48 BALB/c mice were inoculated with 1 × 10^6^ stationary phase *L. major* promastigotes in 50 µl phosphate buffered saline into the Left Hind Footpad (LHFP) using a 29 gauge needle and left for 4 weeks incubation period [34]. The inoculated mice were then randomly assigned into 6 groups of 8 mice in each. Group 1 treated with *A. indica* extracts, group 2 with *R. communis,* group 3 with combination of *A. indica* (10 ml/kg) and *R. communis* (15 ml/kg), group 4 with sterile PBS, group 5 Pentostam and group 6 amphotericin B; treatment commenced on the 5th week post infection (day 29), for 28 days [34–36]. All treatments were done intraperitoneally.

A total of four mice per group were sampled at week 10 for analysis of *L. major* parasite loads. Lesion size, which was defined as the difference in thickness between the infected footpad and the non-infected contralateral Footpad, was monitored weekly by measurement using a Starret dial caliper (Mitutoyo, Suzano, SP, and Brazil) [37, 38]. The weight of the mice was also monitored on a weekly basis. The spleen were removed and weighed and changes post-infection and chemotherapy were determined based on spleno-somatic indices as previously described [39]. Splenic *L. major* burdens were determined from Giemsa-stained impression smears and expressed as Leishman-Donovan units (the number of amastigotes per 1000 host nuclei, multiplied by the weight of the organ); (a) LDU = No. of parasites/1000 host nuclei; (b) Total LDU = LDU × organ weight × 2 × 10^5^ [32, 40, 41].

### Ethical clearance

All procedures were per the Kenya Medical Research Institute (KEMRI) operational and approved protocols under the bioprospecting for plant based anti-leishmanial compounds programme/theme as regulated by the Scientific Steering Committee (SSC), Ethical Review Committee (ERC) and Animal Care and Use committee (ACUC). The guidelines were strictly adhered to during the research.

### Statistical analysis

The data collected on lesion sizes, parasite loads and absorbance were analyzed using the SPSS software. All experiments were performed in triplicate, the mean standard deviation of at least three experiments were determined, statistical analysis of the differences between mean values obtained for the experimental groups was done by the students t test. P values of 0.05 or less were considered to be significant. To determine the efficacy range of the chemical dosages used, a logistical model which can be used to identify non-linear response to ranges of concentrations was fitted to the data. The logit model: logit[θ (x)] = Log[θ^(x)^/1 − θ(x)] = β_0_ + β_1_x_1_ + β_2_x_2_ +…β_i_x_i_ is a general logistic model which takes the form log[ρ/1 − ρ] = β_0_ + β_1_C + β_2_C^2^ + β_3_ + C^3^ in dose response treatments [42]; where ρ denotes the probability of survival, β_0_ is an intercept, β_1_ is the coefficient of concentration C, and β_2_ is the coefficient of quadratic response in C while β_3_ is the coefficient of cubic response in C. All analyzed results were declared significant at *p* < 0.05.

## Results

**Fig. 1:**
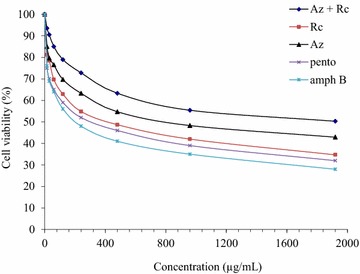
Cell viability of the Vero-E6 cells subjected to the test extracts

Results indicating the cell viability of the Vero-E6 cells subjected to the test drugs are shown in Fig. 1. The Vero-E6 cells were significantly affected by treatment using the test drugs of *A. indica*, *R. communis* and *A. indica* + *R. communis* (*p* < 0.05). The concentration of the test drug required to destroy 50 % of the mammalian cell was significantly low in *R. communis* (92 µg/mL) followed by *A. indica* + *R. communis* (101 µg/mL) and highest in treatment using *A. indica* (149 µg/mL).

**Fig. 2:**
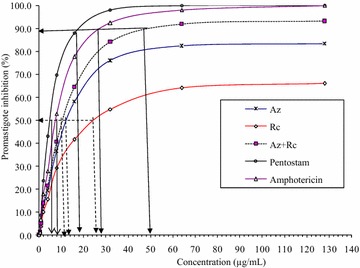
Promastigote growth inhibition following treatments with various test extracts

The promastigotes growth was significantly affected by the various plant extracts (*p* < 0.05) after exposure. The % growth inhibition estimated for the promastigote form of parasite fully fitted the logistic regression model (Table 1). Based on the model parameter coefficients of C, the most effective drug against promastigotes was Amphotericin B followed by Pentostam which are standard drugs for leishmaniasis. Among the extracts the combined therapy of *A. indica* and *R. communis* was found to be the most effective drugs followed by *A. indica* while *R. communis* was the least effective. The efficacy of different concentrations of *R. communis*, *A. indica*, *A. indica* + *R. communis* and standard drugs Pentostam and Amphotericin B on promastigotes of *L. major* is shown in Fig. 2. The optimal efficacy, concentration at optimal efficacy, IC_90_, IC_50_ of the extracts and standard drugs against promastigote forms of the parasite are shown in Table 2. There were significant differences in the optimal efficacy of the test drugs (*p* < 0.05). The optimal efficacy of the standard drugs was 98 and 96.5 % for amphotericin B and Pentostam respectively. Among the test extracts, combined therapy of *A. indica* and *R. communis* was the most effective against promastigote followed by *A. indica* while *R. communis* was the least effective. There was significant (*p* < 0.05) differences in the IC_50_ with the lowest IC_50_ occurring in *A. indica* + *R. communis*, followed by *A. indica* and least in *R. communis* among the known standard test drugs.

**Table 1:** Model parameter statistics from the logistic regression of the five test extracts against promastigotes of *L. major*

Test drug	Model	Parameter significance
*A. indica*	Log (ρ/1 − ρ) = 0.434 + 0.384 × C − 0.0008 × C^2^ + 0.0001 × C^3^	β_0_(0.0000) β_1_(0.0000) β_2_(0.0001) β_3_(0.0026)
*R. communis*	Log (ρ/1 − ρ) = 0.225 + 0.324 × C − 0.0008 × C^2^ + 0.0008 × C^3^	β_0_(0.0000) β_1_(0.0000) β_2_(0.0002) β_3_(0.0123)
Az + Rc	Log (ρ/1 − ρ) = 0.125 + 0.454 × C − 0.0008 × C^2^ + 0.0011 × C^3^	β_0_(0.0000) β_1_(0.0000) β_2_(0.0003) β_3_(0.0016)
AMB	Log (ρ/1 − ρ) = 0.015 + 0.684 × C − 0.0008 × C^2^ + 0.0004 × C^3^	β_0_(0.0000) β_1_(0.0000) β_2_(0.0004) β_3_(0.0021)
Pentostam	Log (ρ/1 − ρ) = 0.012 + 0.724 × C − 0.0008 × C^2^ + 0.0014 × C^3^	β_0_(0.0000) β_1_(0.0000) β_2_(0.0001) β_3_(0.0089)

Az + Rc, combination of *R. communis* and *A. indica*; AMB, amphotericin B

**Table 2:** Optimal efficacy, IC_90_ and IC_50_ of test extracts against promastigote form of the parasite

Concentration (µg/mL)	Test drugs			Controls		Parameter and statistics	
	Az	Rc	Az + Rc	Pentostam	AMB	F value	P value
Optimal efficacy (%)	78	61.5	91.2	96.5	98	26.654	0.002
Concentration at optimal efficacy	43.5	69.5	55.2	30.4	40.2	9.257	0.012
IC_90_	–	–	42	16.2	25.1	15.226	0.003
IC_50_	10.1	25.5	12.2	4.1	5.0	15.456	0.000

Az, *A.indica*; Rc, *R. communis*; Az + Rc, combination of *A. indica* and *R. communis*; AMB, amphotericin B

**Fig. 3:**
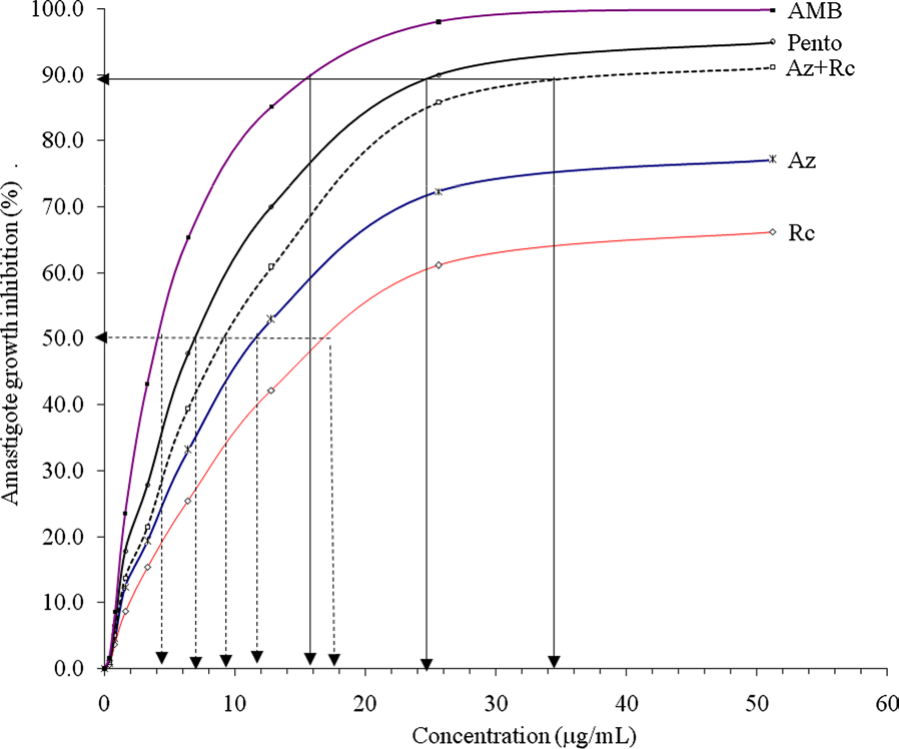
Amastigote growth inhibition following treatments with the test extracts

The efficacy of different concentrations of plant extracts and standard drugs, on amastigotes of *L. major* is presented in Fig. 3. The amastigote growth was significantly affected by the various test plant extracts (*p* < 0.05) of exposure. The % growth inhibition estimated for the amastigote form of parasite fully fitted the logistic regression model (Table 3). Based on the model parameter coefficients of C, the most effective drug was amphotericin B followed by Pentostam, which were standard drugs. Among the test drugs that were used in the current study, the combined therapy of *A. indica* + *R. communis* was found to be the most effective drugs followed by *A. indica* then *R. communis*. The optimal efficacy, concentration at optimal efficacy, IC_90_, IC_50_ of the test drugs against amastigote forms of the parasites are described in Table 4. The optimal efficacy of the extracts was 72, 59.5 and 88 % *A. indica*, *R. communis* and combination of *A. indica* and *R. communis* respectively, compared to standard drugs that had 98 and 92 % for Pentostam and amphotericin B respectively. Only combined therapy of *A. indica* + *R. communis* (34.5 µg/mL) achieved IC_90_ compared to the test drugs which had 15.5 µg/ml, 24.5 µg/ml, for Pentostam and ampotericin B respectively. There was significant (*p* < 0.05) difference in the IC_50_ with the lowest IC_50_ occuring in *A. indica* + *R. communis*, followed by *A. indica* and least in *R. communis* among the known standard test drugs.

**Table 3:** Model parameter statistics from the logistic regression of the plants extracts against amastigote of *L. major*

Test drug	Model	Parameter significance
*A. indica*	Log (ρ/1 − ρ) = 0.434 + 0.384 × C − 0.0008 × C^2^ + 0.0001 × C^3^	β_0_(0.0000) β_1_(0.0000) β_2_(0.0001) β_3_(0.0026)
*R. communis*	Log (ρ/1 − ρ) = 0.225 + 0.324 × C − 0.0008 × C^2^ + 0.0008 × C^3^	β_0_(0.0000) β_1_(0.0000) β_2_(0.0002) β_3_(0.0123)
*Az* + *Rc*	Log (ρ/1 − ρ) = 0.125 + 0.454 × C − 0.0008 × C^2^ + 0.0011 × C^3^	β_0_(0.0000) β_1_(0.0000) β_2_(0.0003) β_3_(0.0016)
AMB	Log (ρ/1 − ρ) = 0.015 + 0.684 × C − 0.0008 × C^2^ + 0.0004 × C^3^	β_0_(0.0000) β_1_(0.0000) β_2_(0.0004) β_3_(0.0021)
Pentostam	Log (ρ/1 − ρ) = 0.012 + 0.724 × C − 0.0008 × C^2^ + 0.0014 × C^3^	β_0_(0.0000) β_1_(0.0000) β_2_(0.0001) β_3_(0.0089)

Az + Rc, Combination of *R. communis* and *A. indica*; AMB, amphotericin B

**Table 4:** Optimal efficacy, IC_90_ and IC_50_ of test extracts against amastigote form of the parasite

Concentration (µg/mL)	Test drugs					Parameter and statistics	
	Az	Rc	Az + Rc	Pento	AMB	F value	p value
Optimal efficacy (%)	72	59.5	88	98	92	17.311	0.002
Concentration at optimal efficacy	25.5	28.2	35.1	25.2	34.5	9.212	0.001
IC_90_	–	–	34.5	15.5	24.5	19.221	0.001
IC_50_	11.5	16.5	9.0	6.5	4.5	12.489	0.000

Az, *A. indica*; Rc: *R. communis*; *Az* + *Rc* combination of *A. indica* and *R. communis*; Pento, pentostam; AMB, amphotericin B

**Fig. 4:**
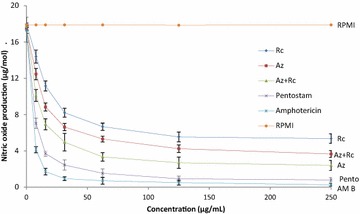
Nitric oxide production in the macrophages of BALB/c infected mice infected with *L. major* and subjected to different treatments by various test extracts

The nitric oxide production in macrophages of BALB/c mice infected with *L. major* amastigotes and subjected to various drugs is shown in Fig. 4. The production of Nitric Oxide decreased in the order *R. communis* > *A. indica* + *R. communis* > *A. indica*, hence treatment with combination therapy leads to more production of NO by the macrophages than that from standard drugs and monotherapy of *A. indica*. The Nitric Oxide produced by macrophages treated with Pentostam and Amphotericin B were the lowest during the experiment. Infected macrophages treated with RPMI produced high amounts of nitric oxide.

**Fig. 5:**
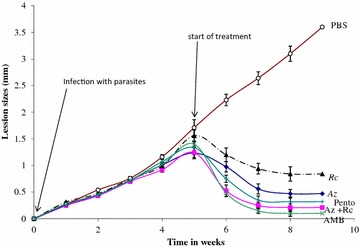
Effect of mono and combination therapy on *L. major*lesion development in BALB/c mice

The lesion sizes of BALB/c mice at the start of infection, during and the start of treatment with plant extracts and controls is shown in Fig. 5. There were no significant differences in development of lesion sizes in BALB/c mice during the first 5 weeks post-infection with *L. major* (*p* > 0.05). Differences in lesion sizes between weeks 5 to week 10 were subjected to repeated measure ANOVA, which indicated that there were significant differences in lesion sizes among different treatments (F = *df* = *p* = 0.05). The lesion sizes of the untreated controls of BALB/c mice increased steadily after infection. Smallest lesion sizes occurred in BALB/c mice treated with combination therapy and amphotericin B, which was slightly lower than BALB/c mice, treated Pentostam. BALB/c mice treated with monotherapies of *A. indica* or *R. communis* had larger lesion sizes. Treatment of BALB/c mice with *R. communis* resulted in the least reduction in lesion sizes among all the tested drugs post treatment. There was no significant difference between the combined therapy and Pentostam (*p* > 0.05); however the monotherapies showed lower activity than standard drugs.

Body weights, weight of spleen, spleen-somatic index and number of parasites in BALB/c infected with *L. major* under various treatments is shown in Table 5. There were significant differences in the weight of spleen, spleno-somatic index and number of parasites among treatments (*p* < 0.05). In *L. major* infected BALB/c mice, the spleen and spleno-somatic index was found to be significantly high when treatment was done using *R. communis* followed by *A. indica.* There was no significant difference between that of combination therapy of *A. indica* + *R. communis* and Pentostam. The index was highest in the untreated controls. BALB/c mice treated with Pentostam and those treated with Amphotericin B, the differences in spleen weight were not significant (*p* > 0.05). The number of parasites was also high in untreated controls, followed by those treated with *R. communis* and *A. indica* while combination treatment with *A. indica* + *R. communis* was lower. Nevertheless, treatment using amphotericin B and Pentostam resulted in the lowest numbers of parasites.

**Table 5:** Body weight, weight of spleen, spleno-somatic index and number of parasites in BALB/c mice following various treatments

Treatment	Body weight	Weight of spleen	Spleno-somatic index	No of parasites
Az	22.11 ± 0.54	0.18 ± 0.021^c^	0.84 ± 0.10^c^	37.3 ± 6.4^c^
Rc	21.78 ± 0.89	0.23 ± 0.021^b^	1.04 ± 0.08^b^	58.4 ± 9.8^b^
Az + Rc	20.50 ± 0.45	0.15 ± 0.006^a^	0.73 ± 0.02^a^	26.4 ± 0.7^a^
Amphotericin B	21.00 ± 1.00	0.13 ± 0.005^a^	0.74 ± 0.06^a^	25.7 ± 0.5^a^
Pentostam	21.00 ± 0.58	0.14 ± 0.010^a^	0.75 ± 0.07^a^	26.1 ± 0.4^a^
PBS	21.00 ± 1.73	0.37 ± 0.014^d^	1.83 ± 0.21^d^	145.7 ± 6.7^d^
ANOVA				
F	2.1332	35.255	71.214	46.987
*df*	5	5	5	5
P	0.3245	0.0001	0.0000	0.0000

Means in the same column followed by the same superscript show no significant difference between them

Az, *A. indica*; Rc, *R. communis*; Az + Rc, *A. indica* and *R. communis* (combination therapy); PBS, phosphate buffered saline (negative control); Pentostam, standard drug (positive control); Amphotericin B, standard drug (positive control)

**Fig. 6:**
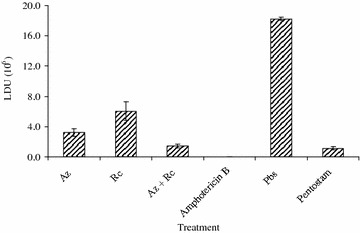
The LDU of *L. major* parasite in spleen of BALB/c mice infected with *L. major* receiving various treatments

There were significant differences in the LDU of *L. major* parasites in BALB/c mice treated with monotherapies of *A. indica*, *R. communis* and a combination of *A. indica* + *R. communis* (ANOVA; F = *df* = P = 0.05). In *L. major* infected BALB/c mice, treatment with Pentostam, amphotericin B and combination therapy resulted in the lowest LDU. The LDU decreased in order of: *R. communis* > *A. indica* > *A. indica* + *R. communis*. There were no significant differences between LDU of BALB/c mice treated with Pentostam and that treated with combination therapy (*p* < 0.05). The LDU of *L. major* parasite in BALB/c mice infected with *L. major* receiving various treatments is shown in Fig. 6.

## Discussion

Chemotherapy against leishmaniasis is mainly based on the antimonial compounds, sodium stibogluconate and meglumine antimoniate (glucantine). The mode of action of antimonials is poorly understood, and -their toxicity causes serious side effects that often result in patients deserting treatment. Furthermore, there is a worldwide escalating frequency of chemo-resistance to antimonials, thus, affordable alternative drugs against leishmaniasis are momentously needed. This study set out to determine the antileishmanial activities of *A. indica* and *R. communis* used in combination and independently against *L. major* infection in susceptible BALB/c mice. A combination therapy of *A. indica* and *R. communis* was very efficient in decreasing lesions in infected BALB/c mice. This antileishmanial activity was compared to that of the standard drugs. The combination therapy was also highly cytotoxic to the promastigotes and amastigotes in vitro while nontoxic to the macrophages and the VeroE6 cells. In vivo, basing on the behaviour of the mice, the extracts were well tolerated during the treatment period.

The monotherapies were initially tested for their antileishmanial activity and the results of this study showed that *A. indica* was of higher potency than *R. communis* however their cytotoxicity against peritoneal macrophages as well as veroE6 cells was still lower than that of the standard drugs. The methanolic extract of *R. communis* reduced *L. major* lesion development, as well as parasite loads in the spleen; this suggests the extract was successful in inhibiting *L. major* parasite growth. Though the activity was lower than that of the standard drugs, this may be attributed to the fact that the extracts in this study were used in their crude form. Studies have shown that isolation of active ingredients of plant extracts improves their potency [43]. If the bioactive phytochemical compounds of *R. communis* that are targeted at *L. major* are isolated while eliminating those that are cytotoxic to body cells it may give different results. This current study is consistent with earlier studies [35, 44] that show potential antimicrobial effect of *R. communis* with evidence of in vivo and in vitro activities of the plant extracts.

Treatment with *A. indica* extracts also exhibited marked reduction in lesion development and parasite load in vitro and in vivo. It is more active against promastigotes than amastigotes but causes production of very low levels of NO from macrophages than that produced by other test compounds. This indicates its low induction activity on peritoneal macrophages for production of NO, than that observed in *R. communis* treatment. Its activity is however better than the standard drugs in term of their cytotoxicity to the macrophages and veroE6 cells. It also fits the logistic regression model for drug administration; which shows its high potential as a candidate for treatment of the *L. major* infection [42]. These findings agree with earlier studies using the same extracts against parasitic organisms of trypanosomatidae [43, 45] and recent work on *L. donovani* [16].

The significant decrease in the lesion sizes in the combined therapy is indicative of the occurrence of a synergistic mechanism between the drug combinations. The two extracts have different modes of action and their combination tends to improve their antileishmanial activity. Moreover, the progression of the splenomegally and the increase of the splenic load were found to be significantly lower in treated mice than in untreated controls, demonstrating parasite suppression and the inhibition of parasite growth. The spleen is a major site of *Leishmania* multiplication in the natural infection in susceptible hosts. In BALB/c mice, the splenic parasite burden is initially quite low, but it increases steadily for at least 3 months, and unlike the hepatic burden, it does not decline spontaneously without treatment [6]. The splenic efficacy of the test compounds should be emphasized, since until recently splenectomy was performed as the last recourse for cases of antimony resistant leishmaniasis. It is evident from the monotherapy results that treatment with either *A. indica* or *R communis* resulted in parasite inhibition but at a lower level than for combination therapy. It can therefore be concluded that administration of the combination of drugs is effective and superior to individual drugs. The synergism shown with the combined drugs brings us to the concept of structure–function approach in fighting leishmaniasis. The effect of combination therapy of *A. Indica* and *R. communis* may be due to complementarity between the two extracts. The standard drugs are known to exhibit cytotoxicity to the cells and cause inflammation at the site of infusion Combination therapy also leads to production of more NO by the macrophages than is seen in the monotherapy of *A. Indica,* thus suggests that the latter treatment up-regulates production of NO by the macrophages and this may be one of the ways in which the parasites are eliminated from the macrophages, Considering that the efficacy towards promastigotes is higher than that against amastigotes, the combined therapy may also have direct cytotoxicity toward the parasites. The penetration of the test compounds into macrophages is hence comparable to that of amphotericin B and poorer than that of Pentostam. Pentostam has been reported to have better action against amastigotes due to its high penetration ability into the macrophages. However, the efficacy of the combination therapy may still be low compared to the standard drugs except for good LDU data, because the extracts were used in their crude form. Using a combination therapy and administration through intra-peritoneal route therefore may have the potential as a good intervention to treatment of cutaneous leishmaniasis in murine models.

Macrophages, the target cells in therapy of leishmaniasis play an important role in the immunological control of intracellular parasites through the production of cytokines and oxygen metabolites [46]. One of the main mechanisms is the up-regulation of nitric oxide inside the macrophages, which is an effective mediator in killing amastigotes [47]. In this study combination therapy caused a slight increase in the NO produced by the macrophages, a slight improvement from that produced by the monotherapies which suggests that it may be one of the modes of action of the extracts but not the only mechanism involved in parasite elimination. This is not seen among the standard drugs. However it is consistent with previous studies that *R. communis* up-regulates production of NO, though in small quantity in the macrophages. *A. indica* may have other mechanism by which it causes its anti-parasitic activity apart from just increasing the amount of NO. This study is in tandem with previous studies that dispute the fact that *A. indica* is capable of activating the immune system to induce production of interferon-gamma (IFN-γ) and tumor necrosis factor-alpha (TNF-α) [48], cytokines that act in synergy in the activation of macrophages to produce nitric oxide and control the infection by *Leishmania* [49]. It is however in agreement with the fact that *A. indica* does not cause up-regulation of NO in the macrophages [43]. The combined therapy however, has a synergy of action leading to its activity, which causes immunomodulatory action. This may have been a point where synergism is well demonstrated by the combined therapy.

The effect of the drugs on the VeroE6 cells in vitro shows that the toxicity of the two extracts is still in the manageable range, this also improves in combination. This was also observed in the in vivo experiments for none of the mice died during the experimental period and there was no marked weight reduction or ill health among the treated mice. This may be due to the fact that for *R. communis*, the leaves are less toxic, have lesser ricin, than the fruits which has been documented to be very toxic [43, 50, 51]. *A. indica* too was only cytotoxic to the parasites but less toxic to the veroE6 cells and the macrophages. The extracts studied showed excellent antileishmanial activity that was unrelated to toxicity, which guarantees safety to the macrophages and specificity to the parasite which is consistent with studies by Nwaka and Hudson [52]. Combining the two drugs does not seem to increase toxicity to veroE6 cells and peritoneal macrophages but improves their potency against the amastigotes and promastigotes. This gives a good base for further investigation to access whether these extracts could become valid candidates for drug development.

There are some limitations associated with this study. First, the in vivo treatment majorly focused on intraperitoneal mode of administration hence other modes of treatment that is intravenous and subcutaneous should also be explored. To confirm on the non-toxic nature of the plant extracts, the effect that various factors such as the growth stage and maturity of the plant, regional variations (where the plant is growing) should be looked into. Leishmaniasis associated cytokine profiles (interferon-gamma (IFN-γ) and tumor necrosis factor-alpha (TNF-α among others) were not analyzed. The evaluation can help give more insight on the synergistic effect of the extracts.

## Conclusion

Results from this study indicates that both plant extracts have antileishmanial activity. The two medicinal extracts when used in combination have synergistic effects in treatment of leishmaniasis. Results of this study also indicated that the test plant extracts had relatively higher cytotoxicity to parasites than the host cells at concentrations used to inhibit growth of the parasites. Based on the observed antileishmanial activity and low or absence of cytotoxicity on the host cells, it is suggested that a combination therapy of *R. communis* and *A. indica* be used for bioassay-guided fractionation, isolation of bioactive compounds and their fortification, which could serve as new drug lead structures. Isolation of bioactive components can also be carried out with the intention of removal of components with strong cytotoxic effects as well as identify and mark those active compounds for antileishmanial activity.
